# Role of Alkyl Hydroperoxide Reductase (AhpC) in the Biofilm Formation of *Campylobacter jejuni*

**DOI:** 10.1371/journal.pone.0087312

**Published:** 2014-01-31

**Authors:** Euna Oh, Byeonghwa Jeon

**Affiliations:** School of Public Health, University of Alberta, Edmonton, Alberta, Canada; East Carolina University School of Medicine, United States of America

## Abstract

Biofilm formation of *Campylobacter jejuni*, a major cause of human gastroenteritis, contributes to the survival of this pathogenic bacterium in different environmental niches; however, molecular mechanisms for its biofilm formation have not been fully understood yet. In this study, the role of oxidative stress resistance in biofilm formation was investigated using mutants defective in catalase (KatA), superoxide dismutase (SodB), and alkyl hydroperoxide reductase (AhpC). Biofilm formation was substantially increased in an *ahpC* mutant compared to the wild type, and *katA* and *sodB* mutants. In contrast to the augmented biofilm formation of the *ahpC* mutant, a strain overexpressing *ahpC* exhibited reduced biofilm formation. A *perR* mutant and a CosR-overexpression strain, both of which upregulate *ahpC*, also displayed decreased biofilms. However, the introduction of the *ahpC* mutation to the *perR* mutant and the CosR-overexpression strain substantially enhanced biofilm formation. The *ahpC* mutant accumulated more total reactive oxygen species and lipid hydroperoxides than the wild type, and the treatment of the *ahpC* mutant with antioxidants reduced biofilm formation to the wild-type level. Confocal microscopy analysis showed more microcolonies were developed in the *ahpC* mutant than the wild type. These results successfully demonstrate that AhpC plays an important role in the biofilm formation of *C. jejuni*.

## Introduction

*Campylobacter jejuni* is a leading bacterial cause of human gastroenteritis and accounts for 400 million–500 million cases of diarrhea worldwide per year [1]. *C. jejuni* is also implicated in the onset of approximately a quarter of cases of Guillain–Barré syndrome, an autoimmune disorder characterized by acute and progressive neuromuscular paralysis [2]. While *C. jejuni* is isolated from a variety of domestic animals and wildlife, poultry is considered to be the major reservoir for *C. jejuni* [3]. *Campylobacter* infection is zoonotic and most cases of human campylobacteriosis are associated with consumption of undercooked poultry [4]. Although *C. jejuni* is known to be fastidious to culture because of complex nutrient and growth requirements [5], this bacterium is isolated from diverse environmental sources, such as surface water, sewage and farms [6], [7], suggesting that *C. jejuni* may possess unique survival mechanisms to persist in the environment. However, mechanisms for stress resistance and survival in the environment have not been well understood in *C. jejuni*.

Oxidative stress resistance is an important defense mechanism. As a microaerophilic bacterium, *C. jejuni* possesses unique oxidative defense systems. *C. jejuni* has a sole catalase (KatA) and a sole superoxide dismutase (SodB) for the detoxification of H_2_O_2_ and superoxide, respectively [8]. In *Escherichia coli*, alkyl hydroperoxide reductase consists of AhpC and AhpF [9] and plays an important role in scavenging endogenous H_2_O_2_ [10], whereas an *ahpF* homolog is absent from the *C. jejuni* genome [11]. Although the substrates of AhpC have not yet been identified in *C. jejuni*, an *ahpC* mutation increases susceptibility to aerobic stress and cumene hydroperoxide, but not to H_2_O_2_ [11]. As to the regulation of oxidative stress response, *C. jejuni* lacks homologs of the oxidative stress regulators OxyR and SoxRS, one or both of which are usually present in many bacterial species [8]. Instead, PerR, Fur and CosR regulate genes of oxidative stress resistance in *C. jejuni* [12]–[15].

Biofilms are a mode of bacterial growth which is often found in natural settings [16]. High numbers of *Campylobacter* spp. are isolated from biofilms in nature [17], implying that the ability of *Campylobacter* to form biofilms contributes to its prevalence in the environment [17]–[19]. *C. jejuni* forms biofilms on various abiotic surfaces, such as glass, plastics and stainless steel [20]–[22]. The biofilm formation of *C. jejuni* is affected by nutritional and environmental conditions. Cultivation with Mueller Hinton media at 37°C under 10% CO_2_ enhanced biofilms, whereas nutrient-rich media (e.g., Brucella broth) or high osmolarity (e.g, >0.05M NaCl) decreases biofilm formation of *Campylobacter* [22]. As motility is an important factor of biofilm formation in many bacterial species [23], mutations of genes associated with bacterial motility significantly affect the biofilm formation of *C. jejuni* [20], [21], [24]. Surface polysaccharides influence biofilm formation as mutations of genes involved in the synthesis of capsular polysaccharide (CPS) or lipooligosaccharide (LOS) increase biofilm formation [20], [25]. Interestingly, the *pgp1* gene encoding a peptidoglycan DL-carboxypeptidase affects the corkscrew morphology of *C. jejuni*, and a *pgp1* mutation results in defects in motility and biofilms [26]. Quorum sensing is also involved in biofilm formation as a *luxS* mutant that is defective in the production of autoinducer-2 (AI-2) exhibited reduced biofilms [22].

Owing to the aggregated bacterial growth in biofilms, bacterial cells in biofilms may encounter a series of nutritional and physiological stress. Thus, bacterial resistance to stress may significantly affect biofilm formation. For example, the stringent response is an important stress resistance mechanism associated with bacterial survival under unfavorable conditions. The stringent response of *C. jejuni* is mediated by SpoT, a bifunctional enzyme that synthesizes and hydrolyzes guanosine teteraphosphate (ppGpp) [27]. In contrast to the stringent response mutants in other bacteria which usually show defects in biofilms, interestingly, the *spoT* mutation significantly increases biofilm formation and produces more mature biofilms compared with the wild type [28]. Oxidative stress resistance significantly impacts *C. jejuni*’s aerotolerance, freeze-thaw resistance, antibiotic resistance, intracellular survival and chicken colonization [11], [14], [29]–[31]. However, nothing is known about the role of oxidative stress resistance in the biofilm formation of *C. jejuni*. In this study, we compared the biofilm formation of mutants defective in key enzymes of oxidative stress resistance, including KatA, SodB, and AhpC, and show that AhpC plays a pivotal role in the biofilm formation of *C. jejuni*.

## Materials and Methods

### Bacterial Strains and Culture Conditions

Bacterial strains used in this study are listed in Table 1. All *C. jejuni* strains were grown at 42°C with Mueller-Hinton (MH) media (Oxoid, Canada) under a microaerobic condition (5% O_2_, 10% CO_2_, and 85% N_2_). Occasionally, MH media were supplemented with kanamycin (50mg/L) and/or chloramphenicol (25mg/L). *E. coli* DH5α harboring plasmids was grown at 37°C with Luria-Bertani (LB) media (Difco, US) that were supplemented with ampicillin (100mg/L), chloramphenicol (25mg/L), or kanamycin (50mg/L), where required.

**Table 1:** Strains and plasmids used in this study.

Strains and Plasmids	Description	Sources or references
*E.coli*		
DH5α	F– Φ80*lac*ZΔM15 Δ(*lac*ZYA-*arg*F) U169 *rec*A1 *end*A1 *hsd*R17 (rK–, mK+)*pho*A *sup*E44 λ– *thi*-1 *gyr*A96 *rel*A1	Life Technologies
*C.jejuni*		
NCTC 11168	Wild type, a human isolate	[37]
Δ*ahpC*	*ahpC* mutant, *ahpC*::*aphA-3*	This study
Δ*katA*	*katA* mutant, *katA*:: *aphA-3*	[31]
Δ*sodB*	*sodB* mutant, *sodB*::*cat*	[31]
Δ*perR*	*perR* mutant, *perR*::*cat*	[57]
*ahpC* over	*C. jejuni* NCTC 11168 harboring an extra copy of *ahpC* in rRNA region	This study
*cosR* over	*C. jejuni* NCTC 11168 harboring an extra copy of *cosR* in rRNA region	[13]
Δ*perR* &Δ*ahpC*	*ahpC* (*ahpC*::*aphA-3*) and *perR* (*perR*::*cat*) double mutant	This study
*cosR* over&Δ*ahpC*	*ahpC* mutant (*ahpC*::*aphA-3*) and CosR- overexpression mutant	This study
*ahpC* comp	*ahpC* complementation	This study
*katA* comp	*katA* complementation	[31]
*sodB* comp	*sodB* complementation	[31]
*perR* comp	*perR* complementation	[57]
Plasmids		
pUC19	Cloning vector used for suicide vector in *C. jejuni*; Amp^r^	New England Biolabs
pFMBcomCM	pUC19 derivative carrying an rRNA gene cluster; Cm^r^	[13]
pMW10	*E.coli-C. jejuni* shuttle vector	[32]

### Construction of the *ahpC* mutant, and the *perR&ahpC* and *cosR* over&*ahpC* double mutants

The *ahpC* gene and its flanking region were amplified by PCR with DahpC-F and DahpC-R primers (Table 2). After digestion with *EcoR*I and *BamH*I, the PCR product was ligated to pUC19 that had been treated with the same enzymes. The pUC19::*ahpC* plasmid was digested with *EcoR*V and ligated with the kanamycin resistance cassette which had been amplified from pMW10 [32] with Kan-F and Kan-R primers (Table 2). The constructed suicide plasmid was introduced to *C. jejuni* NCTC 11168 by electroporation and mutants were selected on MH agar plates supplemented with kanamycin (50 mg/L). The *ahpC* mutation was confirmed by PCR with mahpC-F and mahpC-R primers (Table 2). The *ahpC* complementation strain was constructed by chromosomal integration of *ahpC* as described previously [33]. Briefly, *ahpC* was PCR-amplified with com_ahpC-F and com_ahpC-R primers (Table 2). After digestion with *Xba*I, *ahpC* was ligated with pFMBcomCM [13]. The pFMBcomCM::*ahpC* plasmid was transformed into the *ahpC* mutant strain by electroporation and a complementation strain was selected by growing on MH agar plates containing kanamycin (50mg/L) and chloramphenicol (25mg/L). In addition, pFMBcomCM::*ahpC* was introduced into *C. jejuni* NCTC 11168 as described above to construct an *ahpC*-overexpression strain. Increased transcription of *ahpC* in the *ahpC*-overexpression strain was confirmed by qRT-PCR (Fig. S1). Chromosomal DNA extracted from the *ahpC* mutant strain was introduced into the *perR* mutant and the CosR-overexpression strain by electroporation to construct the *perR*&*ahpC* and *cosR* over&*ahpC* double mutants. Transformants were selected on MH agar plates supplemented with kanamycin (50mg/L) and chloramphenicol (25mg/L). Transfer of the *ahpC* mutation was confirmed by PCR.

**Table 2:** Primers used in this study.

Primers	Sequences (5′-3′)
DahpC-F	GGAATTCCTCCCCACTTCTCATATC
DahpC-R	GGGATCCCAATAGCTGCCGCATCTTG
Kan-F	GCGATGAAGTGCGTAAG
Kan-R	CGGCTCCGTCGATACTATG
mahpC-F	CATGATAGTTACTAAAAAAGCTTTAG
mahpC-R	GTTAAAGTTTAGCTTCGTTTTTGCC
com_ahpC-F	GTCTCTAGAAGCTGCCGCATCTTGAGACTTTG
com_ahpC-R	CGTTCTAGACACCTTCTGGATTGTTAGTATCAT

### Biofilm Assay

Biofilm assay was performed as described previously [25] with some modifications. Briefly, overnight cultures of *C. jejuni* strains were harvested from MH agar plates and resuspended in MH broth to an OD_600_ of 0.07. After culturing 5 h at 42°C with shaking (200rpm) under a microaerobic condition, the bacterial suspension was diluted with fresh MH broth to an OD_600_ of 0.07 and inoculated into 96-well plates (Nunc, US). Biofilms were cultivated under the same growth condition without shaking. The 96-well plates were washed twice with PBS (pH 7.4) and dried 20 min at room temperature after discarding supernatants. Fifty microliter of 1% crystal violet was added to each well. After staining 15 min at room temperature, crystal violet solution was removed and the plates were washed 3 times with PBS (pH 7.4). Stained biofilm was eluted with a solution of 10% acetic acid and 30% methanol, and OD_595_ was measured with a spectrophotometer (Thermo Scientific, US). Occasionally, biofilm assay was carried out in the presence of 1 µM CosR-PNA [34], which was commercially synthesized by PANAGENE (Daejeon, South Korea), or antioxidants, including L-proline, L-cysteine, β-carotene and N-acetyl cysteine, which were purchased from Sigma (St. Louis, US).

### Measurement of Total Reactive Oxygen Species (ROS)

The ROS level in biofilms was measured using CM-H_2_DCFDA (Life Technologies, US), a general oxidative stress indicator. After discarding supernatants from 96-well plates, biofilms were washed twice with PBS (pH7.4) and then treated with 100 µl PBS containing 10 µM CM-H_2_DCFDA for 30 min. Fluorescence was measured with a multi-well plate reader (Varioskan Flash; Thermo Scientific). Protein concentrations of each sample were measured with Bradford protein assay (Bio-Rad, US) to normalize the ROS level.

### Lipid Hydroperoxide (LPO) Assay

LPO levels were measured using a commercial kit (Cayman Chemical Co., US) according to the manufacturer’s instructions. Briefly, LPOs were extracted from biofilms with chloroform and methanol. The LPO extract (500 µl) was mixed with 50 µl of Chromogen reagent (4.5mM ferrous sulfate in 0.1M HCl and 3% methanolic solution of ammonium thiocyanate) and incubated at room temperature for 5 min. A portion (300 µl) of each sample was transferred into a 96-well glass plate to measure OD_500_. A standard curve was generated with 13-hydroperoxy-octadecadienoic acid that was provided by the manufacturer.

### Confocal Microscopy Analysis

Biofilms were fixed with 4% paraformaldehyde and stained with SYTO9 and propidium iodide from the LIVE/DEAD Biofilm Viability kit (Life Technologies, US) according to the manufacturer’s instructions. Confocal microscopy was performed with an inverted confocal microscope (IX-81, Olympus, Japan) and Volocity 3D Image Analysis Software (PerkinElmer Inc., US).

### Statistical Analysis

Data analysis was performed with GraphPad Prism 6 (GraphPad Software Inc., US).

## Results and Discussion

### Enhanced Biofilm Formation in the *ahpC* Mutant

**Figure 1:**
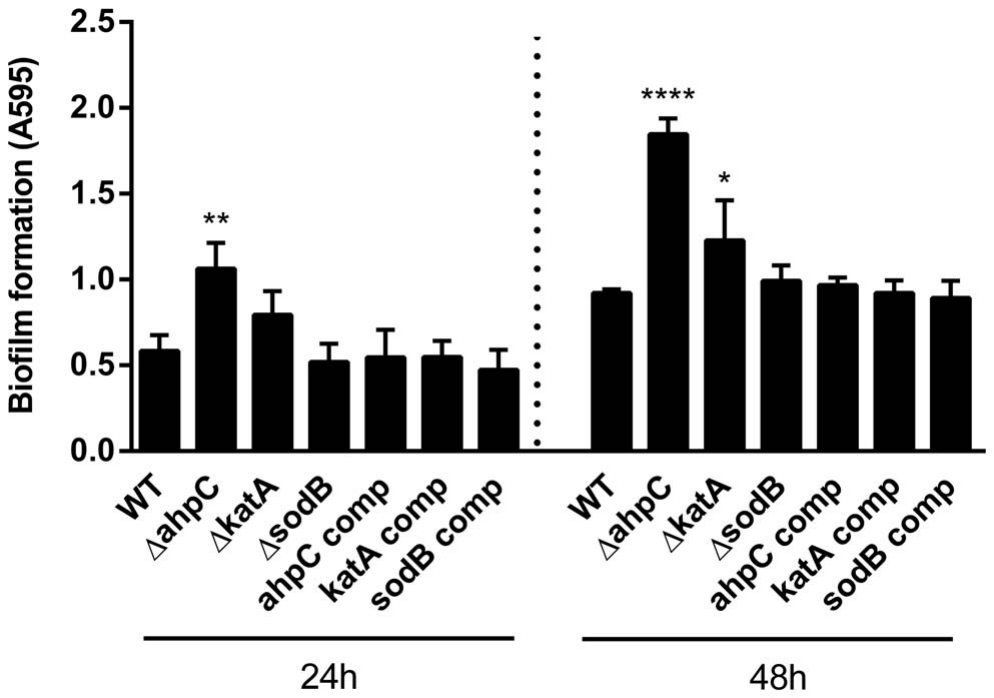
Biofilm formation of oxidative stress resistance mutants. Biofilm formation was measured using crystal violet staining. The results show the means and standard deviations of a representative assay with triplicate samples. The experiment was repeated six times and all produced similar results. Statistical significance was analyzed using one-way analysis of variance (ANOVA). *P≤0.05, **P≤0.01, ****P≤0.0001.

To investigate the role of oxidative stress defense in the biofilm formation of *C. jejuni*, we measured the biofilm formation levels of three oxidative stress resistance mutants defective in AhpC, SodB and KatA. These enzymes were chosen because of their critical roles in oxidative stress resistance, and *C. jejuni* possesses only a single gene copy encoding each enzyme. The *ahpC* mutant displayed most significant increases in the level of biofilm formation compared with the wild type and *katA* and *sodB* mutants, and complementation of the *ahpC* mutant restored biofilm formation to the wild-type level (Fig. 1). The *katA* mutation slightly increased biofilm formation, whereas the *sodB* mutant produced biofilms as comparably as the wild type (Fig. 1). Extended (i.e., 48 h) incubation of biofilms increased the levels of biofilm formation in all the tested strains; however, the *ahpC* mutant developed biofilms to a greater extent than the wild type and *katA* and *sodB* mutants both 24 h and 48 h (Fig. 1). SodB is known to be important for *Campylobacter*’s survival in environmental conditions, such as freeze-thaw stress in foods [35], [36]; however, the *sodB* mutation did not affect biofilm formation (Fig. 1). Bacterial growth is not likely to be associated with the enhanced biofilm formation of the *ahpC* mutant because the *ahpC*, *katA*, and *sodB* mutants grew as comparably as the wild type (data not shown). The *ahpC* mutation slightly reduced the motility (Fig. S2); however, this does not account for the substantial increase in biofilms observed in the *ahpC* mutant, because mutations resulting in a motility defect impair the biofilm formation of *C. jejuni* [20], [21], [24], whereas the *ahpC* mutation substantially increased biofilm formation despite the partial reduction in motility. The results show that AhpC significantly affects the biofilm formation of *C. jejuni*.

### Overexpression of *ahpC* Reduced Biofilm Formation

**Figure 2:**
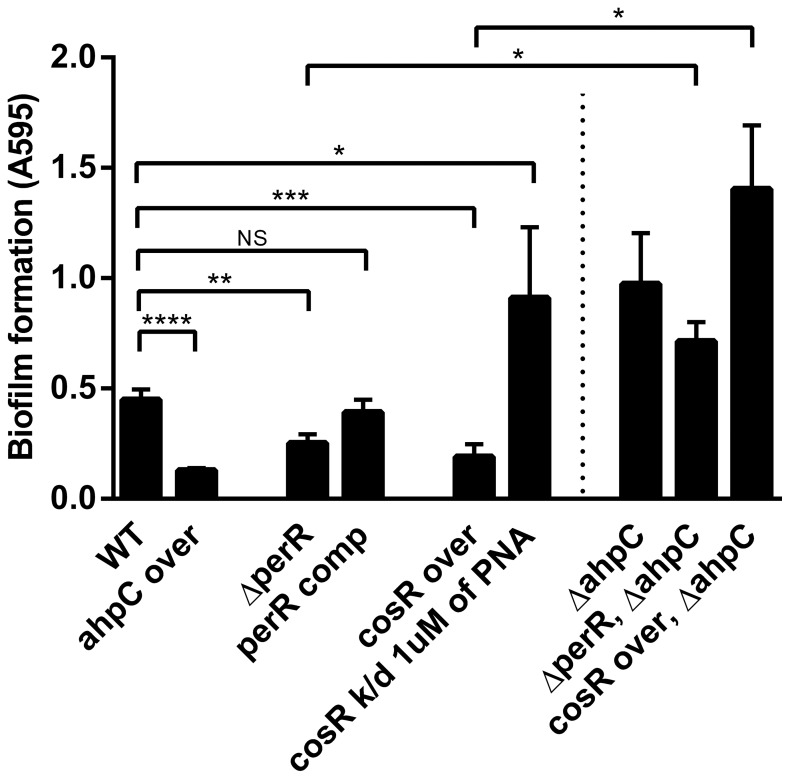
Effect of increased *ahpC* expression on biofilm formation. Biofilm formation levels were determined with crystal violet staining after 24 h incubation of samples. The results show the means and standard deviations of a representative experiment with triplicate samples. The experiment was repeated six times and all produced similar results. The statistical differences between the wild type and each mutant were determined by *t*-test. NS: non-significant, *P≤0.05, **P≤0.01, ***P≤0.001, ****P≤0.0001.

Since AhpC is an enzyme detoxifying ROS, we hypothesized that *ahpC* overexpression would reverse the phonotype observed in the *ahpC* mutant if biofilm formation is directly associated with the enzymatic function of AhpC. The *ahpC* overexpression was achieved in two different ways by: 1) incorporating an extra copy of *ahpC* to the chromosome of *C. jejuni*, and 2) modulating the expression of AhpC-controlling regulators, such as PerR and CosR [15], [34]. *C. jejuni* lacks homologs of OxyR and RpoS [37], which regulate AhpC in *E. coli* and *Salmonella* [38], [39]. Instead, PerR negatively regulates AhpC in *C. jejuni* [15]. CosR is an essential response regulator and positively regulates AhpC in *C. jejuni* [34]. Thus, *ahpC* overexpression was alternatively achieved using a *perR* knockout mutant and a CosR-overexpression strain. In contrast to the stimulated biofilm formation of the *ahpC* mutant (Fig. 1), interestingly, the *ahpC*-overexpression strain exhibited significantly reduced biofilm levels compared to the wild type (Fig. 2), and the *perR* mutant and the CosR-overexpression strain also displayed substantial reductions in the level of biofilm formation (Fig. 2). The *perR* complementation restored biofilm formation to the wild-type level (Fig. 2). CosR is an essential regulator and its knockout mutant cannot be constructed because of bacterial lethality [40], [41]. Thus, a gene knockdown strategy was used to reduce CosR expression with CosR-specific PNA as described in our previous studies [13], [34]. Interestingly, CosR knockdown with CosR-PNA significantly increased biofilm formation to the level of the *ahpC* mutant (Fig. 2).

PerR and CosR are involved in the regulation of genes other than *ahpC* [13], [14], [34]. At least 104 genes belong to the PerR regulon [14], and CosR regulates 93 genes involved in various cellular functions, such as energy production, motility, drug efflux, and oxidative stress resistance [13]. An *ahpC* mutation was introduced to the *perR* mutant and the CosR-overexpression strain to clarify the role of AhpC in the reduced biofilm formation of the *perR* mutant and the CosR-overexpression strain. Interestingly, harboring the *ahpC* mutation abrogated the inhibitory effect of the *perR* mutation and the CosR-overexpression on biofilm formation and substantially increased biofilm formation similar to the *ahpC* mutant (Fig. 2), suggesting that CosR and PerR affect biofilms mainly through *ahpC*.

Kalmokoff et al. showed that proteins of oxidative stress resistance, including AhpC, Tpx (thiol peroxidase), and CosR (Cj0355c), are upregulated in *C. jejuni* biofilms [21]. Consistently, in this study, we demonstrated that both AhpC and CosR significantly affect the biofilm development of *C. jejuni* (Figs. 1 and 2). Given the positive regulation of AhpC by CosR [34], upregulated CosR will increase AhpC expression, consequently promoting the detoxification of ROS in biofilms. Since AhpC overexpression reduced the biofilm formation (Fig. 2), upregulated AhpC will reduce biofilm formation presumably to alleviate oxidative stress in aggregated bacterial cells by decreasing *C. jejuni*’s capability to develop biofilms. Regulators of oxidative stress resistance are also often involved in the modulation of biofilm formation in *C. jejuni*. A mutation of *csrA* (carbon starvation regulator) attenuated both oxidative stress resistance and biofilm formation [42]. CprS is the cognate sensor kinase of the essential response regulator CprR, and the *cprS* mutation enhances biofilm formation and affects the expression of oxidative stress defense proteins, such as AhpC, KatA, CosR, SodB, and TrxB [43]. Based on the previous reports and our findings, the two important defense/survival mechanisms may be functionally related to each other in *C. jejuni*.

### Effect of ROS Accumulation on Biofilm Formation in the *ahpC* Mutant

**Figure 3:**
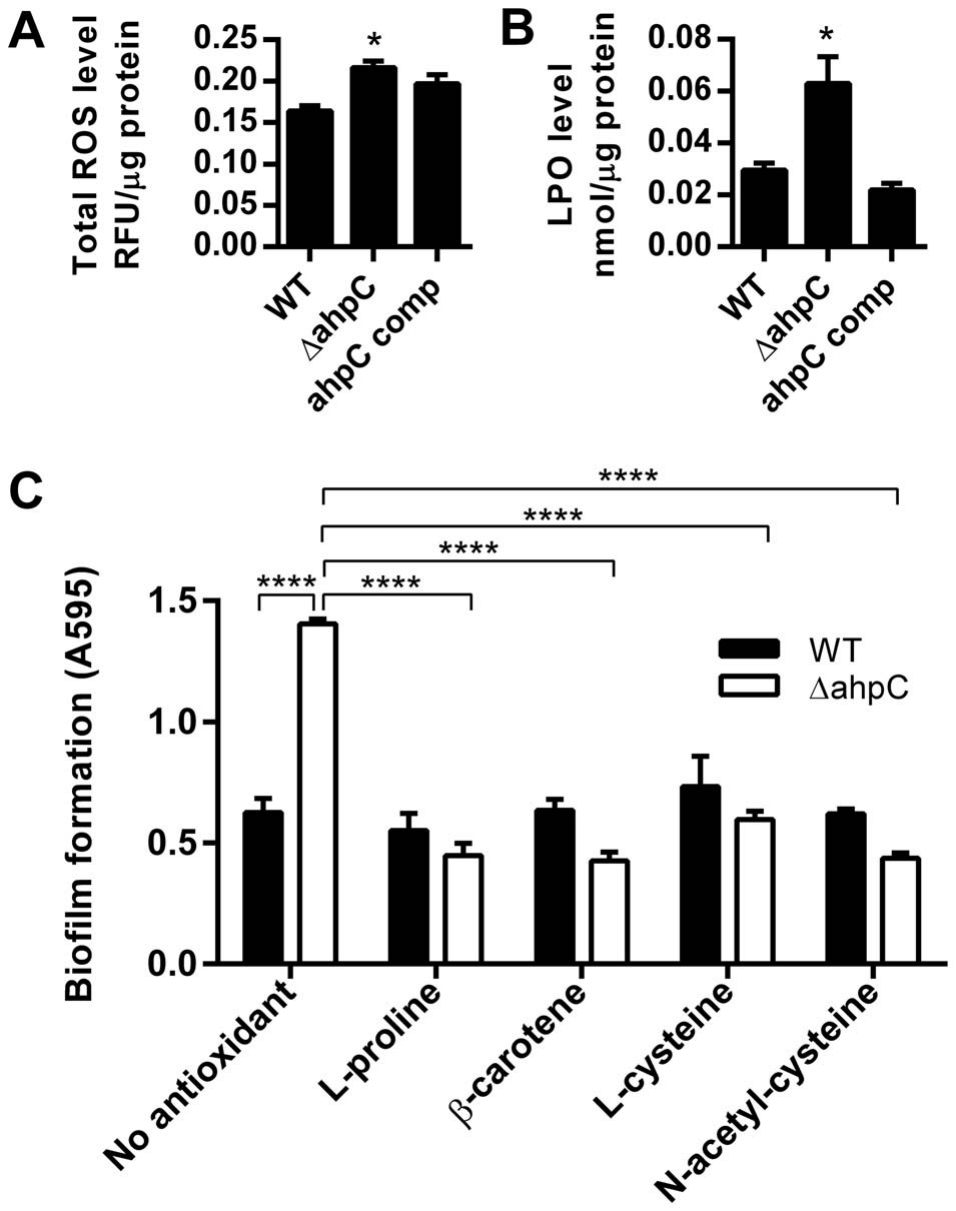
Determination of total ROS level (A) and lipid hydroperoxide (LPO; B) in the *ahpC* mutant, and reduced biofilm formation in the *ahpC* mutant by treatment with antioxidants (C). The assays were carried out with 24 h old samples. Antioxidants were treated to a final concentration of 1nM. The results show the means and standard deviations of a representative experiment with triplicate samples. The assays were repeated at least three times and similar results were reproducible in all the experiments. Statistical significance was analyzed with *t*-test (Fig. 3A and 3B) and two-way ANOVA (Fig. 3C). *P≤0.05, ****P≤0.0001.

AhpC scavenges endogenous H_2_O_2_ at the physiological level in *E. coli* [10]. The substrates of the *Salmonella* Typhimurium AhpC are small hydroperoxides [44] and organic hydroperoxides, including alkyl hydroperoxides which can be produced intracellularly from unsaturated fatty acids and nucleic acids [9]. However, the enzymatic function and substrate of AhpC have not been defined in *C. jejuni*. In this study, total ROS and lipid hydroperoxide (LPO) were measured and compared between the *ahpC* mutant and the wild type (Fig. 3A and B). Consistent with the enzymatic function of AhpC in ROS detoxification, the *ahpC* mutant showed increased accumulation of total ROS and LPO compared to the wild type (Fig. 3A and B). In particular, the LPO level of the *ahpC* mutant was 2-fold higher than that of the wild type (Fig. 3B), suggesting that AhpC may be involved in the detoxification of LPO in *C. jejuni*. The results prompted us to hypothesize that the accumulation of endogenous ROS may be involved in the augmented biofilm formation of the *ahpC* mutant. To examine this possibility, biofilm assay was performed in the presence of antioxidants to reduce the ROS level. Interestingly, addition of antioxidants reduced the biofilm of the *ahpC* mutant to the wild-type level (Fig. 3C). Bacterial growth of the wild type and the *ahpC* mutant was not affected by the treatment with antioxidants (data not shown). The results strongly suggest that the accumulation of ROS enhanced biofilm formation in the *ahpC* mutant.

Bacterial cells in biofilms are physiologically different from planktonic cells and often exhibit increased resistance to environmental stress and antimicrobials [45]. The aggregate growth of bacteria in biofilms reduces the penetration of nutrients into inner layers and limits the diffusion of metabolic wastes, resulting in nutritional limitation and physiological stress [46]. The oxidative stress generated from endogenous ROS in biofilm cells enhances bacterial mutability and diversity [47], [48], demonstrating that ROS substantially impacts bacterial physiology in biofilms. Genes involved in general and oxidative stress response are often induced in biofilms, possibly to alleviate the stress generated in biofilms [21], [48]–[50]. The nutritional starvation of *Pseudomonas aeruginosa* in biofilms confers increased antibiotic tolerance in association with oxidative stress defense as the inactivation of stringent response significantly impaired the activities of catalase and superoxide dismutase [51]. The expression levels of Zn-superoxide dismutase (SodC) and thiol peroxidase (Tpx) are increased in biofilms of *E. coli* O157:H7, and *sodC* and *tpx* mutations impaired biofilm formation [52]. Iron stimulates the formation of rugose biofilms in *E. coli* in connection with ROS. Generation of superoxide stress by adding a superoxide generator or mutating *sodA* and *sodB* promotes the development of rugose biofilms [53]. The biofilm formation of *S. aureus* is stimulated by exposure to cigarette smoke that contains bioactive compounds such as free radicals and ROS. Addition of antioxidant (e.g., N-acetyl cysteine) eliminated the enhanced biofilm formation caused by cigarette smoke exposure [54].

### Enhanced Development of Microcolonies in the *ahpC* Mutant

**Figure 4:**
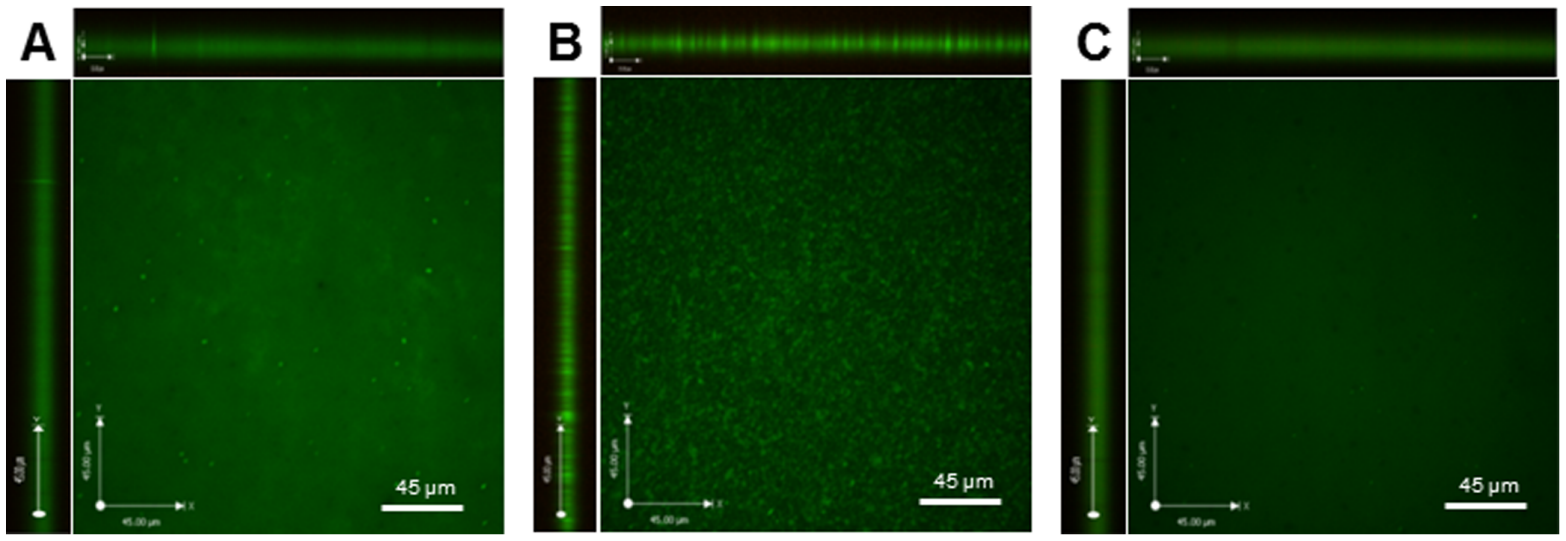
Confocal microscopy analysis of biofilms of the wild type (A), the *ahpC* mutant (B), and the complementation strain (C). Biofilms were grown 24 h and stained with the LIVE/DEAD Biofilm Viability kit (Life Technologies, US).

Biofilm development consists of multiple stages, starting with the attachment of bacteria, formation of microcolonies, maturation of biofilms forming mushroom-like structures and water channels, and dispersion of cells from biofilms [55]. *C. jejuni* forms microcolonies prior to the development of biofilms, and flagellar mutants are defective in forming microcolonies and biofilms [56]. Biofilms were observed with a confocal microscope to investigate the biofilm formation stage that is affected by the *ahpC* mutation. According to the results of confocal microscopy analysis, the *ahpC* mutant showed increased development of microcolonies compared to the wild type (Fig. 4). This is consistent with the results of biofilm assays, showing that the difference in biofilm formation between the wild type and the *ahpC* mutant was obvious even after one day (Fig. 1). The results suggest that AhpC is involved in the development of microcolonies at the early stage of biofilm formation.

Physiological heterogeneity exits in biofilms due to the concentration gradients between layers inside and outside [46]. Reduced diffusion of oxygen into biofilms will expose cells in outside layers to more oxygen and oxidative stress compared to bacteria inside biofilms. The impact of heterogeneity in biofilms would not be significant in the *ahpC* mutant because AhpC affects biofilm formation even at an early stage when biofilms are not fully developed (Fig. 4).

To the best of our knowledge, this is the first report showing that AhpC affects biofilm formation. The kind of ROS responsible for the enhanced biofilm formation of the *ahpC* mutant is still unknown. We speculate that the ROS would be neither H_2_O_2_ nor superoxide, because mutations of *C. jejuni*’s sole catalase and superoxide dismutase did not affect biofilm formation significantly, albeit the *katA* mutant increased biofilm formation at substantially lower levels than the *ahpC* mutant (Fig. 1). Although AhpC scavenges endogenous H_2_O_2_ at physiological levels in *E. coli* [10], such a function has not been demonstrated in the *C. jejuni* AhpC, and addition of exogenous H_2_O_2_ rather reduced the biofilm formation (data not shown). Based on the extrapolation of the enzymatic function of AhpC in other bacteria and the increased accumulation of LPO in the *ahpC* mutant, the ROS involved in biofilm formation in the *ahpC* mutant would be an organic peroxide(s) endogenously generated within the cell. Validation of this possibility first of all requires the identification of ROS substrates of AhpC in *C. jejuni* and still awaits future studies.

## Supporting Information

Figure S1 Increased levels of *ahpC* transcription in the *ahpC* overexpression strain. qRT-PCR was carried out with primer pairs; qPCR_ahpC-F (GGTATTGGTCAGGTTAAATTCCC) and qPCR_ahpC-R (GGTAAATCATTAACCACAGCATG). The results were normalized to the expression level of 16S rRNA (Cjr01) as described previously [34]. The assay was repeated three times. **P≤0.01.
(TIFF)

Figure S2 Motility of the wild type (WT), the *ahpC* mutant (Δ*ahpC*), the *ahpC* complementation strain (*ahpC* comp), and the *ahpC* overexpression strain (*ahpC* over). (A) The *ahpC* mutation resulted in a slight reduction in motility with a full restoration to the wild-type level by complementation. The assay was performed with MH medium containing 0.4% agar, and the motility agar plate was incubated microaerobically at 42°C for 2 days. The result is a representative of three independent experiments with similar results. (B) Comparison of the size of motility zones. The results show the means and standard deviations. NS: non-significant.
(TIF)
