# Supplementary figures and images for: Role of Alkyl Hydroperoxide Reductase (AhpC) in the Biofilm Formation of Campylobacter jejuni
Source: PLoS One. 2014 Jan 31;9(1):e87312. doi: 10.1371/journal.pone.0087312 (PMC3909096; doi:10.1371/journal.pone.0087312)

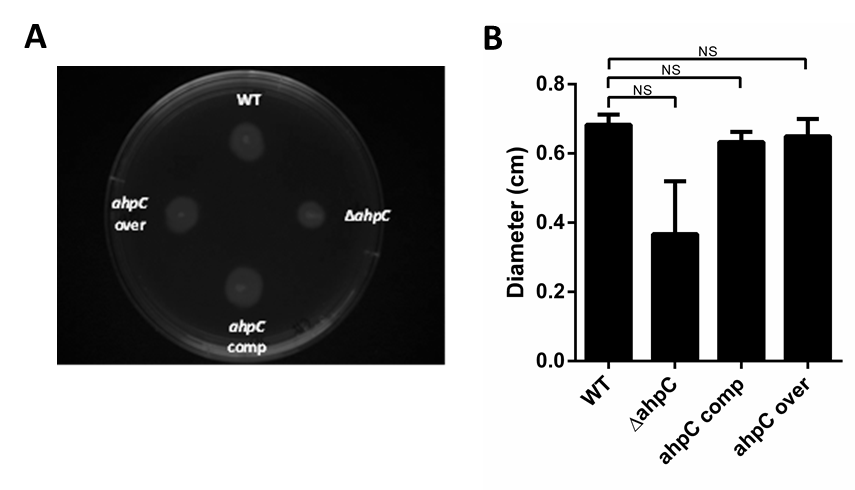

Supplement: Figure S2 — Motility of the wild type (WT), the ahpC mutant (ΔahpC), the ahpC complementation strain (ahpC comp), and the ahpC overexpression strain (ahpC over). (A) The ahpC mutation resulted in a slight reduction in motility with a full restoration to the wild-type level by complementation. The assay was performed with MH medium containing 0.4% agar, and the motility agar plate was incubated microaerobically at 42°C for 2 days. The result is a representative of three independent experiments with similar results. (B) Comparison of the size of motility zones. The results show the means and standard deviations. NS: non-significant. (TIF) [file pone.0087312.s002.tif]
